# Targeting nucleolin to obstruct vasculature feeding with an intelligent DNA nanorobot

**DOI:** 10.1111/jcmm.14127

**Published:** 2018-12-27

**Authors:** Hua Li, Jin Liu, Hongzhou Gu

**Affiliations:** ^1^ Institutes of Biomedical Sciences and Shanghai Institute of Cardiovascular Diseases Zhongshan Hospital Fudan University Shanghai China

**Keywords:** DNA nanorobot, nucleolin, thrombin

## Abstract

A prototype of DNA nanorobot with the ability to transport molecular payloads was designed to target cancer cells in tissue culture. Moreover, a further step was taken to succeed in the first in vivo application of the DNA nanorobot for cancer therapy. The robot was constructed using aptamer and DNA origami to fold a 90‐nm tubular device to carry the blood coagulation protease thrombin inside, shielded from circulating platelets and plasma fibrinogen. The recognition and binding of the aptamer to its tumour‐specific target molecule triggered the robot unfolding to expose thrombin to the blood, which in turn activated coagulation at the local tumour site, resulting in tumour necrosis and inhibition of tumour growth. Since all solid‐tumour feeding vessels are virtually the same, this strategy could be effective against many types of malignant diseases.

## INTRODUCTION

An ideal drug delivery system would be capable of preserving the activity of drug molecules by protecting them against degradation, mitigating toxicity and other biological side effects of drug molecules as well as specifically targeting cells and controlling drug release. So far, a series of nanocarriers, for example, liposomes, cationic dendric polymers, gold nanoparticles and carbon nanomaterials, has been developed for drug delivery in various diseases therapy. However, each of them has some limitation, such as the immune toxicity induced by the heterogeneous size distribution of the liposome carriers, or the safety concerns caused by the toxic elements or residuals of the inorganic nanoparticles.

**Figure 1:**
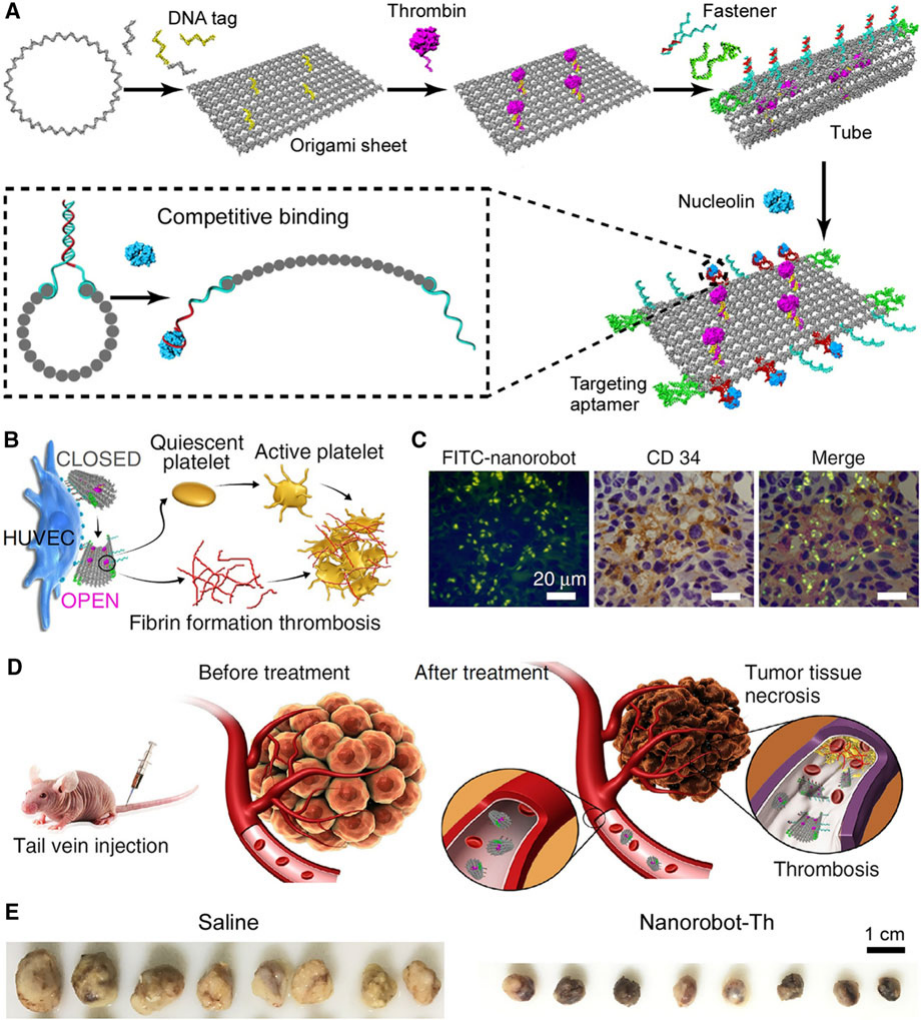
A DNA nanorobot for target therapy. A, Construction of nanorobot‐Th by DNA origami. The robot opened itself upon sensing nucleolin. B, The action mechanism of nanorobot‐Th in plasma with HUVECs. C, Specific targeting of the nanorobot (green) to the blood vessel‐rich regions (brown). FITC‐labelled nanorobots were injected intravenously into mice‐bearing MDA‐MB231 tumours. Tumours were harvested 8 h later, and tumour sections were stained with an anti‐CD34 antibody and examined by confocal microscopy. D, The therapeutic mechanism of nanorobot‐Th within tumour vessels. E, Representative pictures of the tumours from the MDA‐MB231 tumour‐bearing mice after treatment with saline and nanorobot‐Th. Adapted with permission from Reference Li et al.^3^ Copyright 2018 Nature Publishing Group

Fortunately, a promising alternative approach is the use of DNA nanostructures for delivery.^1^ DNA is a genetic material in cells. It is inherently biocompatible and biodegradable. The mature of DNA nanotechnology enables the creation of various 3D nanostructures with precisely controlled size and shape, as well as controllable surface chemistry and dynamic function. The Church lab in 2012 reported a prototype of DNA nanorobot with the ability to transport molecular payloads to targeted cancer cells in tissue culture.^2^ Li et al^3^ took a step further to succeed in the first in vivo application of the DNA nanorobot for cancer therapy (Figure 1). Their robot was constructed using aptamer and DNA origami to fold a 90‐nm tubular device to carry the blood coagulation protease thrombin inside, shielded from circulating platelets and plasma fibrinogen. The recognition and binding of the aptamer to its tumour‐specific target molecule triggered the robot unfolding to expose thrombin to the blood, which in turn activated coagulation at the local tumour site, resulting in tumour necrosis and inhibition of tumour growth. Since all solid‐tumour feeding vessels are virtually the same, this strategy could be effective against many types of malignant diseases.

Developed in 2006,^4^ DNA origami turns out a handy design technique to create arbitrary shapes with a long single‐stranded DNA serving as a scaffold and hundreds of ‘staple’ strands maintaining the scaffold in position. With the origami technique, a 90 × 60 nm rectangular sheet was constructed^3^ (Figure 1A). Through thrombin‐DNA conjugation and DNA‐DNA hybridization, ~4 thrombin payloads on the surface of each DNA sheet were assembled. The sheet was then rolled up to a hollow tube with thrombin attached inside and with a diameter of ~19 nm and a length of 90 nm. Furthermore, “fastener” strands with hybridization were designed to ensure the closure of the tube along the long sides of the sheet. A clever point here was the fastener also serving as a sensor. It contained a DNA aptamer, named AS1411,^5^ which can specifically bind nucleolin – a tumour‐vasculature marker.^6^ With the low level of nucleolin in the blood vessel of healthy tissues, AS1411 was trapped in a DNA duplex by its complementary DNA strand, which together fastened the tube and maintained the conformation. Once entering tumour vasculature where nucleolin was highly expressed on the surface of vascular endothelial cells, AS1411 dissociated from its complementary strand because of the competitive binding to nucleolin, which resulted in the opening of the tube and the exposing of the thrombin cargo. For a better understanding of the working mechanism of the DNA nanorobot, one can imagine that the robot encapsulated the drug molecule thrombin and protected it against non‐specific release when circulating in the blood vessel of healthy tissues. With the molecular sensor – the AS1411 aptamer carried on the robot, as getting into tumour vasculature, the robot recognized the tumour vasculature surface biomarker nucleolin and attached itself to this biomarker to initiate its shape‐shift for targeted exposure of thrombin to the blood.

Although containing multiple components – scaffold, staple, fastener/sensor, payload and even label for imaging, the DNA nanorobot was self‐assembled in one step with a uniform size, which was neat and visualized by atomic force microscopy.^3^ The system was called a robot because it can sense the environmental information and decide when, where and whether to act. The thrombin payload was a serine protease that regulated platelet aggregation by activating platelets and converting circulating fibrinogen into insoluble fibrin, leading to vascular blockage^7^ (Figure 1B). Because of its indiscriminative effects and short circulation half‐life, thrombin had been removed from cancer treatment before. With this DNA nanorobot, to precisely deliver thrombin solely to tumour sites in a highly controlled autonomous manner was managed to maximize its biostability and drug efficacy as well as minimize its toxicity.^3^ The approach in principle was applicable to deliver a wide range of drug candidates that had been previously excluded from clinical use because of unwanted stability issues or side effects.

The nanorobot was confirmed to function well by investigating in vitro blood coagulation (Figure 1B). When they mixed mouse plasma with thrombin‐loaded nanorobot (nanorobot‐Th) and human umbilical vein endothelial cells (HUVECs) which expressed nucleolin on the surface, rapid coagulation was observed. Removing HUVECs significantly slowed down the fibrin formation, indicating that the coagulation was triggered by nucleolin. By further decorating the origami tube at both ends with eight additional AS1411 strands, a maximal targeting effect of nanorobot‐Th to HUVECs was achieved, exhibiting a cell surface dwell time of over 6 hours. When tested on mice‐bearing orthotopic tumours, the nanorobot effectively bound to the tumour vascular endothelium, as evidenced by the co‐localization with the endothelial cell marker CD34 (Figure 1C). Targeted thrombosis in the tumour region after administration of nanorobot‐Th (Figure 1D) occurred within 24 hours, and progressed to a substantial level after 72 hours. No thrombi or histological abnormalities were discovered in the heart, liver, lung, or kidney from the tumour‐bearing mice at all time, indicating that thrombosis was tumour‐vasculature specific. In addition, the nanorobot‐Th exhibited great therapeutic potential to tumours of diverse levels of vascularization, as evidenced by promising in vivo antitumour efficacy in mice‐bearing xenografts of human breast cancer cells MDA‐MB231 (Figure 1E), human melanoma cells B16‐F10 (with high grade of vascularization) and human ovarian cancer cells SK‐OV3 (with low level of vascularization). The observed therapeutic efficacy of this DNA nanorobot‐Th was positively correlated with the vascularization degree of tumours.

Biosafety assessments of the DNA nanorobot system had been carried out.^3^ In non‐tumour‐bearing healthy mice, no significant changes were observed in blood coagulation parameters after treatment with nanorobot‐Th. The robot system also seemed to be immunologically inert in mice and even in healthy Bama miniature pigs which owned the anatomy and physiology closer to humans. This was a good sign for the potential application of the nanorobot system for the treatment of human patients, although human immune responses need to be clarified at the top priority. With a recent biotechnological breakthrough 8 to drastically bring down the cost of folded DNA origami to be around 0.22 $/mg, one major roadblock for clinical test of the nanorobotic system had been cleared. The DNA nanorobot‐controlled vascular occlusion strategy may be more valuable in treating chemoresistant diseases because the robot's functioning mechanism is independent of the special cells’ molecular background, and potential resistance mechanisms that could be developed for the nanorobot‐Th therapeutics are unlikely to overlap with those induced by chemotherapy.^9^ Perhaps, combinations of intelligent nanorobots carrying various therapeutic agents would eventually eradicate malignant diseases and vascularized changes in a well‐orchestrated fashion.

The unique feature of this DNA nanorobot‐Th delivery system is the modularity buried deeply inside DNA nanostructures: The size of a DNA nano‐object and the positions of modifications – ligands, drug molecules, biosensors, etc. – on or within it can be precisely engineered with a nanoscale resolution, and the shape and flexibility of the DNA nano‐object can be fine tuned in vitro and even in vivo by environmental stimulus. It is the modularity that makes this DNA nanorobot a powerful platform to bring in a number of aspects in a controllable manner for targeted drug delivery. Comparing to other drug delivery systems such as liposome and inorganic nanoparticle, the DNA nanorobot system exhibits a few advantages, including low side effects, high delivering specificity, high drug efficacy as well as being able to turn unstable or undrugable molecules into drugable ones. However, before getting into clinical steps, the biodistribution, in vivo stability, translocation through barriers (eg the blood‐brain barrier, the plasma membrane of cell) and the potential immunogenicity of this DNA nanorobot system in patients need to be further checked. Strategies, such as controlling the size of the DNA nanorobot in certain range as well as modifying the surface of the DNA nanorobot with biomimetic materials, can be applied to overcome the potential issues in the above aspects if needed. Despite of the several potential roadblocks for the translation of the DNA nanorobot system from the bench to the bedside, the study of Li et al^3^ definitely is on the right move.

## CONFLICT OF INTEREST

None.
